# Immunological landscape of colorectal cancer: tumor microenvironment, cellular players and immunotherapeutic opportunities

**DOI:** 10.3389/fmolb.2025.1687556

**Published:** 2025-10-30

**Authors:** Adile Buse Andac-Aktas, Gizem Calibasi-Kocal

**Affiliations:** ^1^ Department of Oncology, Institute of Health Sciences, Dokuz Eylul University, Izmir, Türkiye; ^2^ Department of Translational Oncology, Institute of Oncology, Dokuz Eylul University, Izmir, Türkiye

**Keywords:** colorectal cancer, tumor microenvironment, immunotherapy, immunity, immunological heterogeneity

## Abstract

Colorectal cancer (CRC) remains one of the most lethal malignancies worldwide, with outcomes shaped not only by genetic alterations but also by the complexity of the tumor microenvironment (TME). The TME encompasses stromal and endothelial cells, extracellular matrix components, gut microbiota, and a diverse array of immune cells that dynamically interact to influence tumor initiation, progression, and therapeutic response. This review delineates the immunological landscape of CRC, highlighting the dual functions of innate immune cells—including tumor-associated macrophages, natural killer cells, dendritic cells, neutrophils, and mast cells—and adaptive immune players such as cytotoxic T lymphocytes, helper T-cell subsets, and B/plasma cells. These cellular interactions contribute to the heterogeneity between immunologically “hot” microsatellite instability-high (MSI-H) tumors, which are highly responsive to immunotherapy, and “cold” microsatellite-stable (MSS) tumors, which remain resistant. Key mechanisms of immune evasion, such as cancer immunoediting, checkpoint signaling, and exosome-mediated communication, are examined alongside prognostic tools like the Immunoscore that serve as biomarkers of immune infiltration. Emerging immunotherapeutic strategies, including checkpoint blockade, macrophage reprogramming, natural killer cell agonists, and microbiome modulation, are discussed with emphasis on both their promise and limitations in CRC management. By integrating current insights into immune–tumor interactions, the review underscores opportunities for developing personalized, TME-targeted interventions to improve CRC outcomes.

## 1 Introduction

**FIGURE 1:**
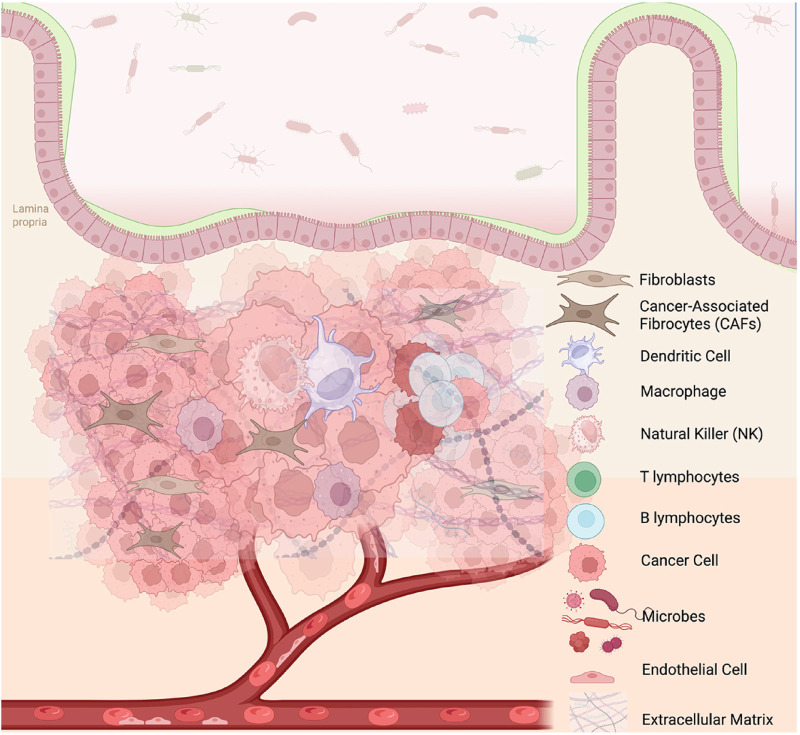
Cellular composition of colorectal cancer microenvironment. This schematic illustrates the complex cellular components within the colorectal cancer microenvironment. The intestinal epithelial barrier and lamina propria are depicted, showing the infiltration of various immune and stromal cell types into the tumor mass. Cancer-associated fibroblasts (CAFs), macrophages, dendritic cells, natural killer (NK) cells, T lymphocytes, B lymphocytes, and fibroblasts are shown interacting with tumor cells. Microbial components from the gut lumen are indicated near the disrupted epithelial barrier, suggesting their potential role in tumor progression and immune modulation. The vascular component highlights the tumor’s access to systemic circulation, which may support metastasis and immune cell trafficking. The image was created with BioRender (https://BioRender.com).

Colorectal cancer (CRC) remains one of the most prevalent malignancies worldwide and represents a major cause of cancer-related mortality (Sung et al., 2021). Despite advancements in screening, surgical techniques, and systemic therapies, many patients progress to metastatic disease, highlighting the necessity for better treatment options (Allemani et al., 2018). Traditionally considered a genetically driven disease, CRC is now increasingly recognized as a complex entity shaped by dynamic interactions between malignant cells and the tumor microenvironment (TME) (Punt et al., 2017) (Figure 1).

The TME comprises a heterogeneous network of stromal components, including fibroblasts, endothelial cells, immune cells, and extracellular matrix (ECM) elements (Baghban et al., 2020; De Visser and Joyce, 2023) (Figure 1). These components not only provide structural support to tumor cells but also actively influence cancer initiation, progression, therapeutic resistance, and metastatic dissemination. Among these, immune cells play a particularly pivotal role by either restraining or promoting tumor development, depending on the balance between anti-tumor immunity and immune evasion mechanisms established by the tumor.

In recent years, growing evidence has highlighted the immunological heterogeneity within CRC tumors and the prognostic and predictive implications of immune cell infiltration patterns (Shembrey et al., 2019; Ferkel et al., 2025). This has led to the emergence of immune-based classification systems, such as the “Immunoscore,” and the increasing interest in immunotherapy for CRC, particularly in subgroups such as microsatellite instability-high (MSI-H) tumors (Gatalica et al., 2016; Hu et al., 2019; Bruni et al., 2020; Galon et al., 2014; Picard et al., 2020).

In this review, we first provide an overview of the CRC microenvironment, followed by a detailed examination of the various immune cell types involved in CRC, including their phenotypic and functional characteristics, their interaction with tumor and stromal cells, and their implications for clinical outcomes and therapeutic response (Pagès et al., 2018; Ciardiello et al., 2019).

## 2 Tumor microenvironment components in colorectal cancer

TME of CRC represents a highly dynamic and complex ecosystem that significantly influences tumor initiation, progression, metastasis, and response to therapy. Comprising a diverse array of cellular and acellular components—including stromal cells, endothelial cells, immune infiltrates, the ECM, and microbial elements—the TME orchestrates continuous interactions that reshape cancer behavior over time (Figure 1) (Pedrosa et al., 2019; Baghban et al., 2020). Understanding the roles and interplay of these components is crucial to identifying novel therapeutic targets and overcoming resistance mechanisms in CRC.

### 2.1 Stromal cells

Stromal cells, particularly cancer-associated fibroblasts (CAFs), constitute a major structural and regulatory component of the CRC microenvironment (Figure 1). These cells facilitate tumor growth by producing ECM components and secreting a variety of soluble mediators, including transforming growth factor-β (TGF-β), interleukin (IL)-6, and hepatocyte growth factor (HGF), which promote cancer cell proliferation, invasion, and immune modulation (Mao et al., 2021). CAFs also upregulate tumor-promoting genes such as CTHRC1, INHBA, BGN, and PDPN, contributing to ECM remodeling and stiffness (Li N. et al., 2023). Recent single-cell transcriptomic analyses show CAFs alone have limited capacity to induce pro-tumorigenic SPP1^+^ macrophages. However, this capacity significantly increases in the presence of tumoroids (Wang et al., 2025).

### 2.2 Endothelial cells

Endothelial cells form the vascular backbone of the TME (Figure 1), supporting tumor growth by facilitating oxygen and nutrient delivery through angiogenesis. These cells are activated by tumor-derived angiogenic factors such as vascular endothelial growth factor (VEGF) and angiopoietins, leading to abnormal vascular networks that also enable immune evasion (Patel et al., 2023; Han et al., 2025). Tumor-associated endothelial cells (TECs) in CRC express immune-modulatory molecules like Fas ligand (FasL) and E-selectin, promoting the exclusion of cytotoxic T cells while facilitating neutrophil recruitment (Motz and Coukos, 2013; Nagl et al., 2020). In transcriptomic datasets, endothelial markers such as CDH5, CLDN5, and ESAM are upregulated in CRC tissues with disrupted tumor architecture (Wang et al., 2019; Tacconi et al., 2015; Liu et al., 2023). CODEX spatial profiling has shown enhanced interaction between TECs and carcinoma cells in high-risk tumors, contributing to the aggressive phenotype of the C3 molecular subtype, which is enriched for angiogenesis-related signatures and portends poor prognosis (Ji et al., 2010; Kuswanto et al., 2023; Cox et al., 2024; Wang et al., 2019; Suo et al., 2025; Schürch et al., 2020). In addition to vascular factors, endothelial cells interact closely with ECM elements to modulate the physical properties of the TME.

### 2.3 Extracellular matrix (ECM)

The ECM provides physical scaffolding and transmits biochemical cues that regulate cell adhesion, proliferation (Figure 1), and migration. In CRC, the ECM is composed of structural proteins such as type I–IV collagens (COL1A1, COL3A1, and COL4A1), fibronectin (FN1), lumican (LUM), and osteonectin (SPARC) (Yue, 2014; Henke et al., 2020; Kim M.-S. et al., 2021). Tumor cells enhance ECM remodeling by upregulating matrix organization genes, which are generally less expressed in tumoroid models, indicating the importance of the *in vivo* microenvironment in ECM dynamics (Page-McCaw et al., 2007; Freitas-Rodríguez et al., 2017). High ECM density and stiffness not only support tumor cell invasion but also serve as physical barriers to immune cell infiltration, facilitating immune exclusion and tumor immune evasion—particularly in the C2 and C3 subtypes (Pickup et al., 2013).

### 2.4 Gut microbiota as a modulator of the TME

The intestinal microbiota plays a critical role in modulating immune responses within the CRC TME (Figure 1). Dysbiosis and the enrichment of pro-oncogenic microbes such as *Fusobacterium* nucleatum, *Bacteroides fragilis*, and *Escherichia coli* can trigger chronic inflammation, activate pro-survival signaling pathways, and modulate immune responses toward tumor tolerance (Xue et al., 2023; Singh et al., 2025). These bacteria contribute to the development of an immunosuppressive TME, further facilitating immune escape (Wong and Yu, 2019). Accordingly, microbiome-targeted strategies such as probiotics, prebiotics, and fecal microbiota transplantation (FMT) are being explored to restore immune homeostasis and improve therapeutic efficacy (Gopalakrishnan et al., 2018; Lei et al., 2025; Mafe and Büsselberg, 2025).

### 2.5 Immune cells

Immune cells are integral to both anti-tumor immunity and tumor progression within the CRC TME (Figure 1). This dual role is shaped by the balance between effector and immunosuppressive cell types. Tumor-infiltrating lymphocytes (TILs), including CD8^+^ cytotoxic T cells, Th1 cells, NK cells, dendritic cells (DCs), and B cells, are associated with improved prognosis and immune surveillance (Kumar et al., 2021; Yu et al., 2025). In contrast, immunosuppressive populations such as regulatory T cells (Tregs), M2-polarized macrophages, myeloid-derived suppressor cells (MDSCs), and SPP1^+^ tumor-associated macrophages (TAMs) support tumor immune evasion and are linked to poor prognosis (Davis et al., 2016; Binnewies et al., 2018; Liu et al., 2025). The immunologically “hot” C4 subtype exhibits a high cytolytic score and enriched effector cell infiltration, in contrast to C2 and C3 subtypes characterized by immune dysfunction and exclusion (Liu et al., 2021; Wu et al., 2023; Xu et al., 2024).

The immune response in CRC is further shaped by the process of cancer immunoediting, which consists of three distinct phases: elimination, equilibrium, and escape. During elimination, immune cells such as CD8^+^ T cells and NK cells identify and destroy tumor cells. If complete elimination is not achieved, the tumor may enter an equilibrium phase where immune pressure selects for resistant clones (Guillerey et al., 2016; Gubin and Vesely, 2022; Tufail et al., 2025). Eventually, immune escape occurs through upregulation of immune checkpoints (e.g., *Programmed cell death protein 1* (PD-1)/Programmed death-ligand 1 (PD-L1), Cytotoxic T-lymphocyte–associated protein 4 (CTLA-4)), secretion of immunosuppressive cytokines, and recruitment of suppressive cell populations. These processes are particularly evident in CRC subtypes with low immunogenicity (C1) and immune dysfunction (C2/C3) (Schreiber et al., 2011; Binnewies et al., 2018). Thus, understanding immune editing processes is fundamental for developing effective immunotherapeutic strategies in CRC.

## 3 Innate and adaptive immune cells in the colorectal cancer microenvironment

The immune response in CRC is further shaped by the process of cancer immunoediting, which consists of three distinct phases Immune cells within the CRC microenvironment represent a diverse and dynamic population that plays a dual role in tumor suppression and promotion. The balance between cytotoxic and immunosuppressive immune subsets determines not only the trajectory of tumor progression but also the patient’s response to immunotherapy. CRC tumors exhibit distinct immunological phenotypes ranging from “hot” tumors, characterized by dense immune infiltration and high cytolytic activity, to “cold” tumors, which are poorly infiltrated and display immune evasion mechanisms (Bonaventura et al., 2019; Wu et al., 2024). These phenotypes often correlate with molecular subtypes—such as the immunologically active C4 and MSI-high tumors versus the immune-excluded C2 and angiogenic C3 subtypes (Wu et al., 2023).

**FIGURE 2:**
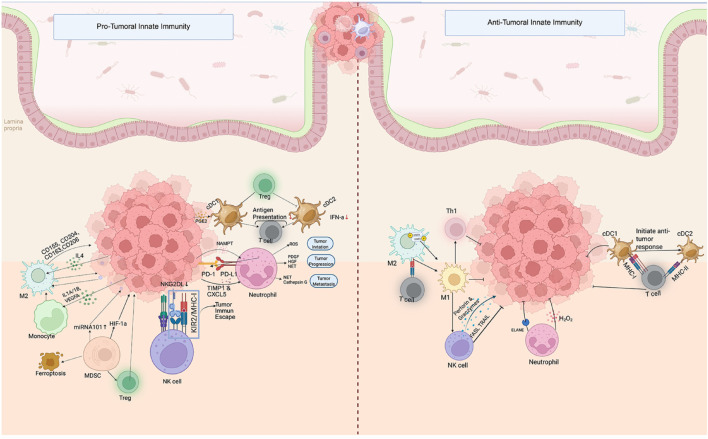
Innate immune cell-mediated pro-tumoral and anti-tumoral activities in the colorectal cancer microenvironment. *Pro-Tumoral Innate Immunity:* Tumor-associated macrophages (M2), myeloid-derived suppressor cells (MDSCs), tolerogenic dendritic cells (DCs), regulatory T cells (Tregs), and neutrophils contribute to immune suppression and tumor progression via mechanisms such as PGE2, IL-10, VEGF, PD-L1 expression, ROS, and NETs. These interactions support tumor initiation, progression, and metastasis while promoting immune escape. *Anti-Tumoral Innate Immunity*: Conversely, classical DC subsets (cDC1/cDC2), M1 macrophages, NK cells, and neutrophils exert anti-tumor activity through enhanced antigen presentation (via MHC-I/II), Th1 polarization, cytotoxic mediator release (perforin, granzyme B, TRAIL), and production of hydrogen peroxide (H_2_O_2_), fostering effective anti-tumor immune responses. The image was created with BioRender (https://BioRender.com).

Immune responses in CRC are shaped by both innate immunity, which provides immediate and non-specific defense, and adaptive immunity, which confers long-term, antigen-specific responses. Key players in the innate compartment include TAMs, dendritic cells (DCs), natural killer (NK) cells, neutrophils, and mast cells (Mantovani et al., 2017) (Figure 2). Adaptive immunity primarily involves CD8^+^ cytotoxic T lymphocytes (CTL), CD4^+^ helper T cells (Th1, Th2, Th17, Tregs), and B cells (Bindea et al., 2013).

### 3.1 Innate immune cells

Innate immune cells serve as the first line of defense against neoplastic transformation, mediating tumor surveillance and shaping subsequent adaptive responses. However, many of these cells are co-opted by tumors to support immunosuppression, angiogenesis, and metastasis (Figure 2).

#### 3.1.1 Tumor-associated macrophages (TAMs)

TAMs are integral components of the innate immune response within the CRC microenvironment, contributing to tumor progression, immune modulation, and therapeutic resistance. Originating from circulating monocytes, TAMs polarize into two functionally distinct phenotypes: pro-inflammatory, anti-tumoral M1 macrophages and immunosuppressive, pro-tumoral M2 macrophages (Figure 2). This polarization is tightly regulated by cytokine signaling, metabolic cues, and tumor-intrinsic factors, with profound implications for CRC progression and patient outcomes (Mantovani et al., 2017; Wang et al., 2021).

M1 macrophages are activated by interferon-gamma *(*IFN-γ), Tumor necrosis factor-alpha (TNF-α), and IL-12, and are characterized by their ability to produce nitric oxide (NO) and reactive oxygen species (ROS), promoting direct tumor cell killing and enhancing Th1 responses through major histocompatibility complex (MHC) class II-mediated antigen presentation (Murray et al., 2014; Chen et al., 2023). These macrophages also activate cytotoxic CD8^+^ T cells via IL-12 secretion, amplifying anti-tumor immunity. High infiltration of M1 macrophages is particularly evident in MSI-H CRC and consensus molecular subtype 1 (CMS1), correlating with robust immune activation, presence of tertiary lymphoid structures (TLS), and favorable prognosis (Roelands et al., 2017; Ferkel et al., 2025). High M1 infiltration is linked to tertiary lymphoid structures (TLS) and improved survival (Shin et al., 2021). Conversely, M2 macrophages promote tissue remodeling, angiogenesis, immune suppression, and metastasis (Figure 2). M2 polarization is induced by IL-4, IL-10, IL-13, and TGF-β, leading to secretion of immunosuppressive cytokines and pro-angiogenic factors such as VEGF and matrix metalloproteinase (MMP)-9 (Gordon and Martinez, 2010; Qian and Pollard, 2010; Lin et al., 2019). In consensus molecular subtype 4 (CMS4) tumors and metastatic disease, high M2 macrophage density is associated with a suppressive TME and poor clinical outcomes (HR = 1.75, 95% CI 1.19–2.58, P = 0.004) (Mlecnik et al., 2016). M2 macrophages facilitate epithelial-mesenchymal transition via CCL-18 and CXCL-12, enhancing tumor invasiveness (Chen et al., 2011; Wang et al., 2021).

The M1/M2 macrophage ratio is a significant prognostic marker. Tumors with a high M1/M2 ratio—frequently MSI-H or early-stage CRC—exhibit improved immune responses and longer survival (HR = 0.58, 95% CI 0.39–0.87, P = 0.008). By contrast, M2-dominated TMEs are linked to increased tumor invasiveness and immune evasion.

M2 macrophages are key facilitators of angiogenesis and metastasis through the production of VEGF-A, VEGF-C, and bFGF, which promote neovascularization (Ferrara et al., 2003; Lewis and Pollard, 2006). Their expression of matrix metalloproteinases (e.g., MMP-2, MMP-9) degrades the ECM, facilitating tumor invasion and metastatic dissemination (Page-McCaw et al., 2007). Additionally, chemokines such as CCL-18 and CXCL-12 produced by M2 macrophages support epithelial-mesenchymal transition and metastatic progression (Wang S. et al., 2024; Zhang et al., 2024).

Polarization is governed by specific signaling cascades: IFN-γ activates STAT1 in M1 macrophages, leading to iNOS upregulation and anti-tumor activity (Mosser and Edwards, 2008), while IL-12 further enhances Th1 and CD8^+^ T cell responses (Henry et al., 2008). In contrast, IL-10 and TGF-β promote M2 polarization via STAT3 and Smad signaling, increasing arginase-1 and PD-L1 expression, suppressing anti-tumor immunity (Flavell et al., 2010; Sica and Mantovani, 2012).

Therapeutically, reprogramming M2 macrophages into an M1 phenotype is a promising strategy. CSF-1R inhibitors, such as PLX3397, deplete M2 macrophages and enhance CD8^+^ T cell activity, improving objective response rates (ORR) in MSI-H CRC (20%–30%) (Shimizu et al., 2024). IL-10 blockade synergizes with PD-1 inhibitors, increasing efficacy in immune-responsive tumors (Pardoll, 2012). Additional approaches include Toll-like receptor (TLR) and CD40 agonists, which potentiate M1 macrophage activation and show promise in early-phase clinical trials (Cheng, 2010). However, their impact remains limited in MSS CRC (Andr’e et al., 2020).

In summary, TAMs exhibit functional plasticity within the CRC TME, with M1 macrophages promoting tumor elimination and M2 macrophages fostering progression and immune evasion (Figure 2). Understanding TAM polarization dynamics and targeting the M2 phenotype are crucial for enhancing immunotherapeutic outcomes and personalizing treatment strategies in CRC (Table 1).

**TABLE 1:** Table summarizes the distinct functions, prognostic impacts, and clinical significance of M1 and M2 macrophages in colorectal cancer, highlighting their roles in anti-tumoral immunity versus tumor progression.

Macrophage type	Functions	Impact on CRC prognosis	Clinical significance
M1 Macrophages	NO/ROS production, antigen presentation, Th1 response via IL-12	High infiltration linked to better overall survival	Prognostic marker; predicts ICI response; target for TLR/CD40 agonists
M2 Macrophages	VEGF/MMP-9 production IL-10/TGF-β-mediated immunosuppression, epithelial-mesenchymal transition promotion	High infiltration linked to poor prognosis	Target for CSF-1R inhibitors, IL-10 blockers; associated with metastasis

Abbreviations in alphabetical order: IL: interleukin, MMP: matrix metalloproteinases, NO: nitric oxide, ROS: reactive oxygen species, TGF-β: Transforming growth factor-β, Th1: T helper 1 cells, TLR: Toll-like receptor, VEGF: vascular endothelial growth factor.

#### 3.1.2 Natural killer (NK) cells

Natural killer (NK) cells are pivotal effectors of the innate immune system in the CRC microenvironment, exerting direct cytotoxic effects on tumor cells and orchestrating broader immune responses. Unlike cytotoxic T lymphocytes, NK cells can recognize and kill transformed cells independently of MHC class I expression, making them essential in countering tumor immune evasion. However, the CRC TME—especially in microsatellite stable (MSS) and consensus molecular subtype 4 (CMS4) tumors—is characterized by immunosuppressive factors such as TGF-β and PD-L1, which impair NK cell function and facilitate tumor progression. Understanding NK cell dynamics in the TME and developing strategies to restore or enhance their function are critical for effective immunotherapy, particularly in immunologically “cold” tumors.

NK cells eliminate tumor cells via direct lysis and modulate the adaptive immune system through cytokines such as IFN-γ and TNF-α. They are regulated by a balance of activating receptors (e.g., NKG2D, DNAM-1) and inhibitory receptors (e.g., KIRs, NKG2A) (Lanier, 2005; Vivier et al., 2011). High NK cell infiltration, especially in MSI-H tumors and early-stage CRC, correlates with favorable overall survival (HR = 0.59, 95% CI 0.38–0.92, P = 0.019) (Sconocchia et al., 2014). In CMS1 CRC (14% of cases), NK cells contribute to a robust immune milieu characterized by abundant cytotoxic T cells and tertiary lymphoid structures (TLS) (Guinney et al., 2015; Roelands et al., 2017). IFN-γ from NK cells promotes dendritic cell maturation and Th1 polarization, enhancing anti-tumoral immunity (Böttcher et al., 2018).

NK cells are uniquely effective in eliminating tumor cells with reduced or absent MHC class I, consistent with the “missing-self” hypothesis. They achieve this through NKG2D-mediated recognition of stress ligands such as MICA/B and ULBPs (Kärre et al., 1986; Lanier, 2005). In MSI-H CRC, high MICA expression enhances NK cell-mediated cytotoxicity and is associated with better prognosis (Lanuza et al., 2022). Conversely, NK cell infiltration is diminished in metastatic and CMS4 CRC (1.5% vs. 5.2%, p < 0.01), correlating with adverse outcomes (HR = 1.82, 95% CI 1.20–2.76, P = 0.005) (Tosolini et al., 2011).

Beyond direct cytotoxicity, NK cells mediate antibody-dependent cellular cytotoxicity (ADCC) through Fcγ receptor III (CD16), especially when engaged by monoclonal antibodies such as cetuximab targeting EGFR (Van Cutsem et al., 2009). NK cells also induce apoptosis via FasL and TRAIL death receptor pathways (Smyth et al., 2003). However, their activity is curtailed in MSS CRC and immunosuppressive TMEs enriched with PD-L1 and TGF-β (Mlecnik et al., 2016).

TGF-β is a principal inhibitor of NK cell function, reducing NKG2D and NKp46 expression and IFN-γ production via Smad signaling (Tauriello et al., 2018; Bruno et al., 2019; Chaudhry et al., 2022). TGF-β is overexpressed in CMS4 and metastatic CRC and is linked to poor clinical outcomes (HR = 1.78, 95% CI 1.15–2.75, P = 0.009) (Tauriello et al., 2018). PD-L1 on tumor and immune cells suppresses NK activity through PD-1 engagement, dampening cytotoxicity (Quatrini et al., 2020).

Additional inhibitory influences include IL-10, hypoxia, and metabolic stress. IL-10 suppresses NK proliferation and IFN-γ production via STAT3 activation (Witalisz-Siepracka et al., 2022; Wang et al., 2023). Hypoxia-driven HIF-1α signaling diminishes NKG2D ligand expression and impairs cytotoxicity (Baginska et al., 2013), while acidic, lactate-rich TMEs suppress NK cell metabolism and effector function (Husain et al., 2013).

Therapeutic strategies targeting NK cell activation aim to counteract immunosuppression and boost antitumoral responses. IL-15 enhances NK proliferation, survival, and IFN-γ production (Waldmann, 2003), with IL-15 super agonists (e.g., ALT-803) showing promising results in MSI-H CRC (Objective response rate-ORR 25%–35%, PFS 8–10 months) (Wrangle et al., 2018; Andr’e et al., 2020). While IL-2 is effective at low doses when combined with immune checkpoint inhibitors (ICIs) (ORR 20%–30%) (Rosenberg, 2014), its toxicity at high doses remains a limitation.

ICIs such as anti-PD-1 (e.g., pembrolizumab) restore NK activity and elicit high response rates in MSI-H CRC (ORR 33%–55%) (Andr’e et al., 2020). Anti-TGF-β agents block Smad signaling, upregulating NKG2D and enhancing NK responses (PFS 10–12 months in early trials) (Alshahrani et al., 2024; Deng et al., 2024). Inhibitors of NKG2A (e.g., monalizumab) lift NK cell suppression mediated by HLA-E, with phase I/II trials showing encouraging results (ORR 20%–25%) (André et al., 2022). Experimental strategies such as CAR-NK cells and bispecific antibodies targeting NK receptors and tumor antigens are also under active investigation (Guillerey et al., 2016). However, therapeutic efficacy in MSS CRC remains limited (ORR 5%–10%), reinforcing the need for subtype-specific approaches.

In summary, NK cells are indispensable in the innate immune landscape of CRC, particularly in MSI-H tumors where they mediate effective immune surveillance and contribute to favorable outcomes. Their functional impairment in immunosuppressive TMEs necessitates targeted interventions. Understanding NK cell receptor signaling, tumor immune evasion strategies, and the molecular driver of suppression is essential for the development of NK-based immunotherapies tailored to CRC subtypes.

#### 3.1.3 Dendritic cells

Dendritic cells (DCs) serve as critical antigen-presenting cells within the CRC microenvironment, bridging innate and adaptive immunity. They play a pivotal role in priming and activating tumor-specific T cell responses by processing and presenting tumor antigens via MHC class I and II molecules, thereby initiating cytotoxic CD8^+^ T cell and helper CD4^+^ T cell responses (Banchereau and Steinman, 1998; Hou et al., 2024).

In immunologically active CRC subtypes, particularly MSI-H tumors, DCs are frequently enriched and are associated with high cytotoxic T cell infiltration and the presence of tertiary lymphoid structures (TLS), correlating with improved overall survival (HR = 0.62, 95% CI 0.41–0.94, P = 0.024) (Mlecnik et al., 2016; Hou et al., 2024). Through secretion of cytokines such as IL-12 and IFN-α, DCs support Th1 polarization and natural killer (NK) cell activation, thereby amplifying anti-tumoral immunity (Trinchieri, 2003).

However, in immune-excluded or immunosuppressive CRC subtypes such as MSS and CMS4, DCs are functionally impaired. The TME inhibits DC maturation via TGF-β, IL-10, and VEGF, skewing them towards a tolerogenic phenotype that promotes immune tolerance rather than tumor elimination. This immature or tolerogenic DCs exhibit diminished antigen-presenting capacity, reduced co-stimulatory molecule expression, and increased PD-L1 expression, leading to T cell anergy and limited efficacy of ICIs (Gabrilovich, 2004; Worthington et al., 2012; Hato et al., 2024).

Low DC infiltration and functional impairment in CMS4 CRC are associated with poor prognosis (HR = 1.75, 95% CI 1.18–2.60, P = 0.005) (Guinney et al., 2015; Guo et al., 2020). While DC-based immunotherapies, such as DC vaccines, have demonstrated encouraging results in MSI-H CRC (PFS 10–12 months in phase I/II trials), their efficacy in MSS tumors remains limited (5%–10% ORR) (Saxena et al., 2021). Advancing our understanding of DC functional states and TME interactions is vital for designing DC-targeted strategies that can overcome immunosuppressive barriers and enhance anti-tumor immunity in CRC.

#### 3.1.4 Neutrophils and mast cells

Neutrophils and mast cells are key myeloid components of the CRC microenvironment that contribute to both pro- and anti-tumoral processes, with their effects modulated by disease stage, molecular subtype, and the immune contexture of the TME.

Neutrophils exhibit functional plasticity in CRC. In early-stage or MSI-H CRC, they can exert anti-tumoral effects via secretion of ROS and TNF-α, directly damaging tumor cells. Their infiltration is modestly associated with improved overall survival in these settings (HR = 0.71, 95% CI 0.48–1.05, P = 0.089) (Fridlender et al., 2009; Rao et al., 2012). However, in advanced and MSS CRC, neutrophils predominantly promote tumor progression (Takesue et al., 2020; Huang et al., 2024). Elevated neutrophil-to-lymphocyte ratio (NLR) is a robust negative prognostic biomarker (Dell’Aquila et al., 2018).

Pro-tumoral neutrophil functions include the formation of neutrophil extracellular traps (NETs), which facilitate tumor invasion, metastatic niche formation, and immune evasion. NETs are associated with liver metastases in CRC (p = 0.002) and decreased disease-free survival (Demkow, 2021). NETs contribute to angiogenesis by releasing VEGF and IL-8 and are enriched in CMS4 tumors, which display immunotherapy resistance (ORR 10%–15%) (Huyghe et al., 2020; Lee et al., 2020; Mousset et al., 2023).

Mast cells, while less studied, are emerging as important regulators of angiogenesis and immune modulation in CRC. They release pro-angiogenic factors including VEGF, histamine, and IL-8, enhancing tumor vascularization and progression (Maltby et al., 2009). High mast cell infiltration in CMS4 CRC correlates with angiogenic and immunosuppressive TME features and poor prognosis (HR = 1.65, 95% CI 1.10–2.47, P = 0.015) (Omatsu et al., 2023). Nonetheless, mast cells may also support anti-tumor immunity by secreting IL-9 and TNF-α, particularly in MSI-H settings (Sinnamon et al., 2008).

Targeting NETs and mast cell-derived mediators offers therapeutic potential in CRC. DNase I disrupts NETs and, when combined with ICIs, shows enhanced efficacy (PFS 8–10 months, preclinical) (Park et al., 2016). Understanding the context-specific roles of neutrophils and mast cells is essential to unlocking new therapeutic strategies aimed at overcoming immunotherapy resistance and modulating the CRC immune landscape (Table 2).

**TABLE 2:** The functions, prognostic implications, and clinical relevance of neutrophils, myeloid-derived suppressor cells (MDSCs), mast cells, and dendritic cells in colorectal cancer, emphasizing their dual roles in tumor suppression and promotion.

Cell type	Functions	Impact on CRC prognosis	Clinical significance
Neutrophils	NET formation, VEGF/IL-8 production, ROS-mediated tumor lysis	High NLR linked to poor prognosis	Prognostic marker; target for NET inhibitors
MDSCs	T cell suppression, IL-10/TGF-β production	High infiltration linked to poor prognosis	Immunotherapy resistance; target for PDE-5/anti-GM-CSF therapies
Dendritic Cells	Antigen presentation, Th1 response via IL-12	High infiltration linked to better overall survival	DC vaccines; ICI response prediction
Mast Cells	VEGF/histamine production, IL-9-mediated immune activation	High infiltration linked to poor prognosis	Angiogenesis-targeted therapies

Abbreviations in alphabetical order: DCs: Dendritic cells, GM-CSF: Granulocyte-macrophage colony-stimulating factor, ICIs: Immune checkpoint inhibitors, IL: interleukin, MDSCs: Myeloid-derived suppressor cells, NET: neutrophil extracellular trap, NLR: Neutrophil-to-lymphocyte ratio, PDE-5: Phosphodiesterase type 5, ROS: reactive oxygen species, TGF-β: Transforming growth factor-β, Th1: T helper 1 cells, VEGF: vascular endothelial growth factor.

### 3.2 Adaptive immune cells

**FIGURE 3:**
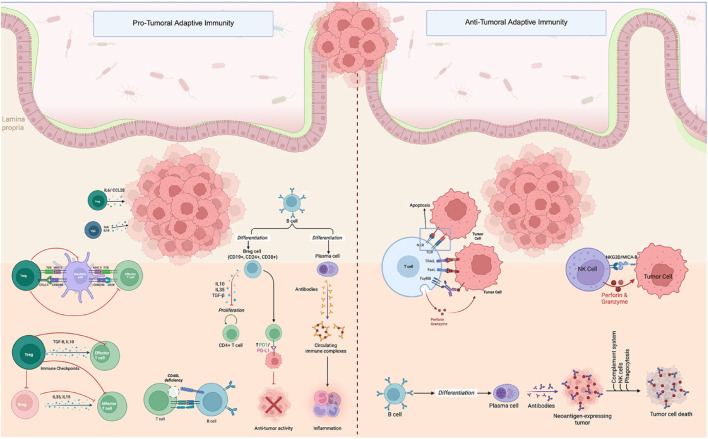
Adaptive immune cell-mediated pro-tumoral and anti-tumoral activities in the colorectal cancer microenvironment. *Pro-Tumoral Adaptive Immunity:* Regulatory T cells (Tregs), regulatory B cells (Bregs), and tolerogenic dendritic cells inhibit anti-tumor responses via checkpoint molecules (CTLA-4, PD-1), immunosuppressive cytokines (IL-10, IL-35, TGF-β), and downregulation of effector T cell activity. Bregs contribute to inflammation and immune complex formation through abnormal antibody production and impaired CD4^+^ T cell proliferation. *Anti-Tumoral Adaptive Immunity:* Effector CD8^+^ T cells and NK cells recognize and eliminate tumor cells via MHC-TCR interaction, death ligands (FasL, TRAIL), and cytotoxic granules (perforin, granzyme). Activated B cells differentiate into plasma cells, producing tumor-specific antibodies that facilitate immune-mediated tumor clearance through complement activation, phagocytosis, and ADCC. The image was created with BioRender (https://BioRender.com).

Adaptive immunity plays a central role in orchestrating tumor-specific immune responses and ensuring long-term immune surveillance in CRC (Figure 3) (Bruni et al., 2020). The adaptive immune compartment is primarily composed of T lymphocytes and B cells, which recognize tumor-associated antigens through highly specific antigen receptors. These cells mediate anti-tumor effects through cytokine secretion, direct cytotoxic activity, and the establishment of immunological memory. Among them, CD8^+^ cytotoxic T lymphocytes (CTLs) and CD4^+^ helper T cells are key players in coordinating effective immune responses. However, in many CRC subtypes—particularly those with low tumor mutational burden or enriched in immunosuppressive factors—adaptive immune function is often compromised. The density, spatial distribution, and activation status of adaptive immune cells within the TME have emerged as strong prognostic indicators and form the foundation for immune-based classification (scoring) systems such as the “Immunoscore (Galon et al., 2014).”

#### 3.2.1 CD8^+^ cytotoxic T lymphocytes (CTLs)

CD8^+^ CTLs are essential components of the anti-tumor immune response in CRC, functioning through recognition of tumor antigens presented via MHC class I molecules (Figure 3). Upon antigen engagement through the T cell receptor (TCR), activated CTLs release cytolytic molecules such as perforin and granzymes, inducing caspase-mediated apoptosis in tumor cells. They also secrete cytokines like IFN-γ, TNF-α, and IL-2, which promote MHC class I expression and recruit additional immune effector cells via chemokines including CXCL9, CXCL10, and CXCL11 (Fridman et al., 2017; Farhood et al., 2019; Huff et al., 2019; Li et al., 2021).

High infiltration of CTLs in both the tumor core and invasive margins is associated with improved overall survival (OS) and reduced recurrence in CRC patients (Reissfelder et al., 2015; Barbosa et al., 2021; Shen et al., 2022). These observations have led to the development of the Immunoscore, which quantifies CD3^+^ and CD8^+^ T cell densities and has demonstrated superior prognostic value compared to conventional TNM staging (Galon et al., 2014; Bruni et al., 2020; Mlecnik et al., 2023).

The strength and efficacy of CD8^+^ T cell responses are shaped by the tumor’s immunogenicity. MSI-H tumors, which account for approximately 15% of stage II and 4% of metastatic CRC cases, harbor high neoantigen loads and are more immunogenic. These tumors typically exhibit abundant CTLs infiltration and maintain cytolytic activity, particularly in the CMS1 and immune-inflamed C4 molecular subtypes, correlating with favorable prognosis and robust responses to ICIs (Le et al., 2015; Llosa et al., 2015; André et al., 2022).

In contrast, MSS tumors often show limited CD8^+^ T cell infiltration and are less responsive to immunotherapy. This immune resistance is frequently compounded by an immunosuppressive TME, where dense ECM and soluble factors hinder effective T cell access and function (Bao et al., 2020; Borràs et al., 2023; Tang et al., 2025). Moreover, persistent antigen stimulation, hypoxia, and metabolic stressors within the TME contribute to T cell exhaustion, a dysfunctional state marked by high expression of inhibitory receptors such as PD-1, Lymphocyte-activation gene 3 (LAG-3), and T-cell immunoglobulin and mucin-domain containing-3 (TIM-3), and a decline in cytotoxic capacity (Jiang et al., 2015; Thommen and Schumacher, 2018; Franco et al., 2020; Tsui et al., 2022).

To reverse T cell exhaustion and reinvigorate anti-tumor immunity, ICIs targeting PD-1 and CTLA-4 have been successfully employed, particularly in MSI-H CRC patients. Agents like nivolumab (anti-PD-1) and ipilimumab (anti-CTLA-4) have demonstrated durable clinical responses in this subgroup. Additional immunotherapeutic strategies under investigation include adoptive transfer of tumor-infiltrating lymphocytes (TILs) and tumor antigen-specific vaccines designed to boost CTLs function (Dudley et al., 2008).

However, the immunologically “cold” nature of many MSS tumors necessitates combination therapies aimed at improving T cell infiltration and activity. Approaches involving chemotherapy, anti-angiogenic agents (e.g., VEGF inhibitors), or ECM-targeting strategies are being explored to overcome immune exclusion and enhance ICI responsiveness (Liu and Sun, 2021; Melssen et al., 2023; Quan et al., 2023).

In summary, CD8^+^ T cells are central to immune-mediated tumor control in CRC, particularly in immunologically active subtypes. Yet, the functional impairment of these cells within suppressive TMEs remains a major therapeutic barrier. Future research should prioritize elucidating the mechanisms underlying T cell exhaustion and developing combinatorial strategies to enhance CTL-mediated immunity across diverse CRC subtypes.

#### 3.2.2 CD4^+^ helper T cells

CD4^+^ T cells are pivotal regulators of adaptive immunity in CRC, exhibiting both anti-tumor and pro-tumor functions depending on their subset differentiation and local microenvironmental cues (Toor et al., 2019; Aristin Revilla et al., 2022; Li et al., 2025; Revilla et al., 2025). Upon antigen recognition via major histocompatibility complex (MHC) class II molecules on antigen-presenting cells (APCs), naïve CD4^+^ T cells differentiate into specialized effector subsets—Th1, Th2, Th17, and regulatory T cells (Tregs)—each governed by distinct transcriptional regulators and cytokine profiles (Figure 3) (Tosolini et al., 2011).

Th1 cells, directed by T-bet and STAT4, secrete IFN-γ, TNF-α, and IL-2, enhancing antigen presentation, promoting M1 macrophage polarization, and recruiting cytotoxic CD8^+^ T lymphocytes (Mlecnik et al., 2016; Orecchioni et al., 2019). IFN-γ also exerts direct anti-tumoral effects by inducing tumor apoptosis through Bcl-2 downregulation and impairing angiogenesis via CXCL-10 induction. Th1 cell predominance is observed in MSI-H and CMS1 tumors, correlating with enhanced immune activation and favorable responses to ICIs.

In contrast, Th2 cells, driven by GATA3, produce IL-4, IL-5, and IL-13, facilitating M2 macrophage polarization and fostering an immunosuppressive TME (Gao et al., 2022; Han et al., 2023; Xu et al., 2025). Although Th2-mediated eosinophil recruitment may occasionally support anti-tumor responses, their overall effect is tumor-promoting, primarily through suppression of Th1 responses. Th2 enrichment has been associated with poorer outcomes, particularly in CMS2 and CMS3 subtypes.

Th17 cells, defined by RORγt expression and stabilized by IL-6, IL-21, and IL-23, secrete IL-17A, IL-17F, and IL-22. These cytokines drive angiogenesis, enhance stemness and chemoresistance, and attract immunosuppressive immune subsets. While IL-17A is largely associated with tumor progression and metastasis—especially in the aggressive CMS4 subtype—IL-17F may exhibit anti-tumor activity in early-stage CRC by supporting cytotoxic T cell function. The dualistic nature of Th17 cells underscores the context-dependent complexity of their role in CRC) (Ouyang et al., 2008; Romagnani et al., 2009; Amicarella et al., 2017; Anvar et al., 2024).

Regulatory T cells (Tregs), characterized by FOXP3 expression and induced by TGF-β and IL-2, are essential for maintaining immune tolerance. They suppress anti-tumor immune responses through secretion of IL-10, IL-35, and TGF-β, and via expression of immune checkpoint molecules such as CTLA-4 and LAG-3. In CRC, Treg accumulation can signal either suppression of tumor-promoting inflammation or facilitation of immune escape, depending on tumor subtype and TME context. Notably, in CMS4 tumors, high Treg infiltration has been linked to poor prognosis (Saito et al., 2016; Tanaka and Sakaguchi, 2017). Further highlighted the prognostic significance of Treg heterogeneity, identifying FOXP3^+^ subsets with divergent effects on clinical outcomes.

The distribution and function of CD4^+^ T cell subsets are dynamically shaped by hypoxic stress, lactate accumulation, and cytokine-mediated signaling within the TME. These factors can skew CD4^+^ T cell polarization toward either effector or regulatory phenotypes, thereby influencing immune surveillance or evasion. From a clinical perspective, robust Th1 responses are predictive of favorable outcomes and sensitivity to immunotherapies such as anti-PD-1 and anti-CTLA-4 antibodies, which restore effector function and reduce Treg-mediated suppression. In MSI-H CRC, these therapies have achieved objective response rates (ORR) up to 40%–55%, while in other subtypes, resistance remains a challenge (Caza and Landas, 2015; Overman et al., 2018; Vito et al., 2020; Martínez-Méndez et al., 2022; Andreu-Sanz and Kobold, 2023).

Emerging therapeutic strategies targeting CD4^+^ T cells include blockade of IL-17 signaling to counteract Th17-driven tumor progression, and the development of CD4^+^ T cell-based vaccines and adoptive T cell therapies. However, these approaches remain largely experimental and require further clinical validation.

In conclusion, CD4^+^ T cells constitute a functionally heterogeneous population within the CRC microenvironment. While Th1 cells bolster anti-tumor immunity and improve clinical outcomes, Th2, Th17, and Tregs often support tumor progression through immune suppression or inflammatory signaling. Understanding the plasticity and interplay of these subsets is critical for designing effective immunotherapeutic strategies and tailoring treatment to individual CRC immune profiles.

#### 3.2.3 B Cells and plasma cells

B cells contribute to tumor immunity through antigen presentation, cytokine production, and antibody secretion (Figure 3) (Shimabukuro-Vornhagen et al., 2014; Wouters and Nelson, 2018). Tumor-infiltrating B cells can support T cell responses but may also have regulatory functions depending on their activation status. Plasma cells, terminally differentiated B cells, have been observed in tertiary lymphoid structures (TLSs) within CRC tumors, particularly in MSI-H and inflamed phenotypes. Their presence has been linked to enhanced immune surveillance and better response to immunotherapy.

B lymphocytes are essential components of the adaptive immune response within the CRC microenvironment, displaying context-dependent anti-tumoral or pro-tumoral effects. Their functions include antibody production, antigen presentation, cytokine secretion, and chemokine-mediated immune cell recruitment. Key subpopulations—such as tumor-infiltrating B cells (TiBc), tumor-associated B cells (TABs), and tumor-associated plasma cells (TAPCs)—modulate tumor progression and clinical outcomes by shaping the immunological landscape of CRC.

The impact of B cell infiltration on CRC prognosis is highly context specific. High infiltration of CD20^+^ B cells, especially in MSI-H and right-sided colon tumors, is associated with improved overall survival (OS) (HR = 0.53, 95% CI 0.36–0.78; multivariable analysis HR = 0.51, 95% CI 0.33–0.80, P = 0.025) (Berntsson et al., 2016; Edin et al., 2019; Xia et al., 2023). This beneficial effect is most prominent in early-stage and immunologically “hot” tumors, particularly in CMS1 CRC (Edin et al., 2019). CD20^+^ B cells contribute to anti-tumoral responses via antibody secretion (IgA, IgG, IgM), antigen presentation to CD4^+^ T cells, and the formation of tertiary lymphoid structures (TLS) that support local adaptive immunity (Wouters and Nelson, 2018). TLS—composed of follicular dendritic cells, T follicular helper (Tfh) cells, B cells, and plasma cells—form near invasive tumor margins and are orchestrated by signals such as LTα/β, CXCL13, and CCL21, and are strongly associated with improved disease-free survival and response to ICIs (Teillaud et al., 2024; Jammihal et al., 2025).

Despite these beneficial roles, certain B cell subsets exert immunosuppressive effects. Regulatory B cells (Bregs; CD24^high^CD38^high^) produce IL-10 and TGF-β, dampening CD8^+^ T cell responses and promoting tumor immune evasion in CMS4 CRC (Schwartz et al., 2016; Sarvaria et al., 2017; Liu et al., 2020; Catalán et al., 2021; Tan et al., 2022). In metastatic CRC, a decline in total B cell infiltration (2% vs. 6.9%, p < 0.05) is accompanied by an increase in Bregs (6.3% vs. 1.1%, p < 0.05) (Maus et al., 2014; Schwartz et al., 2016; Xu et al., 2022). CMS4 tumors (23% of CRCs) exhibit high Breg content and the poorest prognosis (OS: HR = 1.67, 95% CI = 1.34–2.06) (Tosolini et al., 2011; Ten Hoorn et al., 2022). Additionally, plasma cell marker CD138 expression on tumor cells correlates with poor prognosis (HR = 1.52, 95% CI 1.03–2.24) (Berntsson et al., 2016), highlighting the duality of humoral immunity in CRC. Some studies, suggest a non-significant survival benefit from high B/plasma cell scores (P = 0.08), reflecting a neutral effect in certain contexts (Zhang Z. et al., 2019; Karjalainen et al., 2023; Sirkiä et al., 2025).

Mechanistically, B cells recognize tumor antigens via the B cell receptor (BCR), supported by co-stimulatory molecules like CD40/CD40L and cytokines (e.g., IL-4, IL-21), which drive activation and differentiation into memory B cells, plasma cells, or Bregs. BCR engagement activates pathways including NF-κB and PI3K/AKT, regulating survival, proliferation, and antibody secretion (Kurosaki, 1999; Burger and Wiestner, 2018; Michaud et al., 2021; Wolf et al., 2022). Plasma cells (CD138^+^) secrete high levels of IgA, IgG, and IgM (Blanc et al., 2016; Meylan et al., 2022). B cells mediate anti-tumor activity through (Laumont and Nelson, 2023):

- *Opsonization:* Antibodies (especially IgA and IgG) bind tumor antigens, tagging cells for phagocytosis—an effect pronounced in MSI-H tumors (Kinker et al., 2021; Fridman et al., 2022).
- *Antibody-Dependent Cellular Cytotoxicity (ADCC):* Fcγ receptor-mediated activation of NK cells and macrophages by IgG antibodies induces tumor cell lysis (Clynes et al., 1998). Cetuximab enhances this response, increasing ORR to 20%–30% in metastatic CRC (Van Cutsem et al., 2009).
- *Antigen presentation:* MHC class II-mediated activation of CD4^+^ T cells by B cells, particularly within TLS, amplifies adaptive responses through CXCL13 and CCL19 (Kinker et al., 2021).

In contrast, Bregs suppress anti-tumor immunity via IL-10 and TGF-β production, activating the JAK/STAT3 and Smad pathways to inhibit cytotoxic T cell and dendritic cell functions, while PD-L1^+^ B cells contribute to immune checkpoint-mediated resistance (Zhang et al., 2019b; Catalán et al., 2021; Li T. et al., 2023).

The diversity of tumor-associated B cells (TABs), as revealed by single-cell RNA sequencing, reflects a spectrum from IFN-γ-activated anti-tumor phenotypes to IL-10^+^ immunosuppressive profiles (Schwartz et al., 2016; Li M. et al., 2023; Yang Y. et al., 2024). TAPCs, particularly in MSI-H CRC, are associated with favorable humoral responses and TLS density, yet their function may be suppressed by TGF-β and IL-10 in CMS4 tumors (Baker et al., 2009; Lin et al., 2020; Küçükköse et al., 2022).

TME components dynamically shape B cell activity. Hypoxia activates HIF-1α, limiting antibody production and enhancing IL-10 expression. Acidosis and lactate accumulation impair BCR signaling. Stromal fibroblasts and MDSCs modulate B cell differentiation via CXCL12, IL-10, and TGF-β (Xiong et al., 2023; Li et al., 2024; Yang H. et al., 2024).

Clinically, B cells offer diagnostic, prognostic, and therapeutic utility. High CD20^+^ B cell and TLS densities predict improved overall survival and disease-free survival (Wouters and Nelson, 2018; Fridman et al., 2022; Laumont et al., 2022; Laumont and Nelson, 2023). Anti-PD-1 therapies (e.g., pembrolizumab) enhance IFN-γ-producing B cells and TLS formation, showing 33%–55% ORR in MSI-H CRC (André et al., 2022). Monoclonal antibodies like cetuximab boost IgG-mediated ADCC, while IL-10 inhibitors and B cell vaccines are under early-phase evaluation (Zahavi and Weiner, 2020; Azadi et al., 2021; Hwang et al., 2021; Zhou et al., 2024).

In conclusion, B cells and plasma cells are immunologically multifaceted players in CRC. Their roles range from promoting effective humoral and cellular anti-tumor responses to driving immunosuppression via Bregs. Therapeutic modulation of B cell functions, particularly in MSI-H and CMS1 tumors, offers promising avenues for biomarker development and personalized immunotherapy in CRC.

### 3.3 Functional interplay and immune editing in CRC

The role of individual immune cells and their interactions with each other, as well as the mechanisms of immune regulation, are all critical in CRC. The complex interactions between immune cells and tumor cells within the CRC microenvironment play a pivotal role in shaping tumor progression, immune regulation, and the effectiveness of immunotherapeutic interventions. This dynamic crosstalk involves immune checkpoint signaling, cytokine and chemokine networks, and extracellular vesicles such as exosomes, which together modulate the balance between anti-tumor immunity and immune evasion.

#### 3.3.1 Immune checkpoint molecules and tumor escape mechanisms

Immune checkpoint pathways are crucial regulators of T cell activation and represent a major mechanism of tumor immune escape (Kalbasi and Ribas, 2020; Zhou et al., 2020; Dutta et al., 2023; Lin et al., 2024). Tumor cells exploit molecules like PD-1, CTLA-4, and LAG-3 to suppress cytotoxic immune responses. PD-1, expressed on activated T cells, binds PD-L1 on tumor or immune cells, leading to T cell exhaustion (Pardoll, 2012; Qin et al., 2019; Tang et al., 2022). While MSI-H CRC tumors typically express high PD-L1 and show favorable responses to ICIs (ORR 33%–55%), MSS CRCs exhibit low PD-L1 expression and are associated with poor ICI responsiveness (ORR 5%–10%) (Qin et al., 2019; Rotte, 2019).

CTLA-4 competes with CD28 for CD80/CD86 binding on antigen-presenting cells and suppresses early T cell activation. Anti-CTLA-4 therapy (e.g., ipilimumab) enhances T cell responses but is largely limited to MSI-H tumors (Overman et al., 2018). LAG-3 suppresses CD4^+^ T cell activity via MHC class II interaction and contributes to immune tolerance. Emerging inhibitors such as relatlimab show potential in combination regimens (ORR 20%–25%, phase I/II) (Nagasaki et al., 2020; Huo et al., 2022). Tumor-secreted cytokines like TGF-β and IL-10 amplify immune checkpoint-mediated suppression (Chen and Mellman, 2017). In CMS4 CRC, high expression of checkpoint molecules is linked to adverse prognosis (Tosolini et al., 2011).

#### 3.3.2 Cytokines and chemokines in immune modulation

Cytokines like TGF-β, IL-10, and IL-6 orchestrate immunosuppressive networks within the TME. TGF-β activates Smad signaling, inhibits effector T cell proliferation, induces Tregs and M2 macrophages, and facilitates tumor invasion (Calon et al., 2012; Tauriello et al., 2018). Elevated TGF-β correlates with ICI resistance and poor outcomes (HR = 1.78, 95% CI 1.15–2.75, P = 0.009). IL-10, via STAT3, impairs T and NK cell responses and expands MDSCs and Tregs. IL-6 drives tumor proliferation and angiogenesis and contributes to MDSC recruitment and therapy resistance (Dahmani and Delisle, 2018; Kim B.-G. et al., 2021; Mirlekar, 2022; Peng et al., 2022). Chemokines such as CCL2 and CXCL8 attract immunosuppressive myeloid populations, while CXCL9/10 promote CTLs infiltration and are linked to improved prognosis in MSI-H CRC (Chen et al., 2019; Yang et al., 2021; Hu et al., 2025).

#### 3.3.3 Extracellular vesicles (exosomes) in immune editing

Exosomes are critical mediators of intercellular communication in CRC. Tumor-derived exosomes carry PD-L1, TGF-β, and immunomodulatory microRNAs that inhibit T cell responses and promote immunosuppressive cell recruitment (Olejarz et al., 2020; Raimondo et al., 2020; Hao et al., 2022; Sheta et al., 2023). Elevated exosomal PD-L1 correlates with poor outcomes in metastatic CRC (Ayala-Mar et al., 2021; Xian et al., 2021; Yin et al., 2022). Exosomes can also be immunostimulatory: dendritic cell-derived exosomes present tumor antigens and enhance ICI efficacy in MSI-H CRC (Kugeratski and Kalluri, 2021; Yin et al., 2022; Lu et al., 2024). Conversely, CMS4 tumors secrete exosomes rich in miR-21 and miR-155, which suppress NK activity (Kotelevets and Chastre, 2023). Novel therapeutic approaches utilizing engineered exosomes (e.g., anti-PD-L1-loaded) have shown preclinical efficacy in reversing immune evasion (Poggio et al., 2019).

Taken together, the interplay between immune checkpoints, cytokine gradients, and exosomal signaling orchestrates immune editing in CRC. These mechanisms underlie both immune evasion and therapeutic responsiveness, especially in MSI-H versus MSS subtypes. Strategic targeting of these pathways—particularly in MSS CRC—holds promise for overcoming immunotherapy resistance and optimizing personalized treatment strategies (Table 3). These immune cell profiles and molecular subtypes directly impact prognosis and treatment strategies.

**TABLE 3:** Table highlights the mechanisms, prognostic impacts, and clinical significance of interactions between immune cells and tumor cells, including immune checkpoint inhibitors, cytokines, and exosomes, in colorectal cancer.

Mechanism	Functions	Impact on prognosis	Clinical significance
Immune checkpoint molecules	T cell suppression via PD-1/PD-L1, CTLA-4, LAG-3; promotes tumor immune escape	High PD-L1/LAG-3 expression linked to poor prognosis in CMS4/C3 subtypes	ICI therapies (e.g., anti-PD-1: pembrolizumab, ORR 33%–55% in MSI-H; anti-CTLA-4: ipilimumab; anti-LAG-3: relatlimab, ORR 20%–25%)
Cytokines	Immunosuppression (TGF-β, IL-10, IL-6); Treg/M2 induction; tumor growth	High TGF-β1/IL-6 levels correlate with poor outcomes and ICI resistance	Anti-TGF-β (e.g., galunisertib), anti-IL-6 (e.g., siltuximab), and JAK/STAT3 inhibitors under investigation
Chemokines	Immunosuppressive cell recruitment (CCL2, CXCL8); CTLs infiltration (CXCL9/10)	CXCL9/10 associated with improved prognosis in MSI-H/C4	Chemokine-modulating therapies (e.g., CCL2 inhibitors) to reduce MDSC recruitment; CXCL9/10 agonists to enhance CTLs infiltration
Exosomes (Tumor-Derived)	Carry PD-L1, TGF-β, miR-21/miR-155; suppress T/NK cells; promote MDSCs/Tregs	High exosomal PD-L1 linked to poor prognosis in metastatic CRC.	Anti-PD-L1-loaded engineered exosomes; exosome-based diagnostics for immunotherapy resistance
Exosomes (DC-Derived)	Present tumor antigens; stimulate effector T cell responses	Enhanced ICI efficacy in MSI-H CRC	DC-derived exosome vaccines to boost anti-tumor immunity in combination with ICIs

Abbreviations in alphabetical order: CCL2: C-C motif chemokine ligand 2, CMS: consensus molecular subtype, CRC: colorectal cancer, CTLA-4: Cytotoxic T-lymphocyte–associated protein 4, CTLs: Cytotoxic T lymphocytes, CXCL8: C-X-C motif chemokine ligand 8, DCs: Dendritic cells, ICIs: Immune checkpoint inhibitors, IL-6: Interleukin-6, LAG-3: Lymphocyte-activation gene 3, M2: Tumor-associated macrophages, MDSCs: Myeloid-derived suppressor cells, MSI-H: Microsatellite instability-High, ORR: objective response rate, PD-1: Programmed cell death protein 1, PD-L1: Programmed death-ligand 1, TIM-3: T-cell immunoglobulin and mucin-domain containing-3, TGF-β: Transforming growth factor-β, Treg: Regulatory T cell.

## 4 Immunological prognosis and therapeutic modulation in CRC

Immune cells act as key prognostic indicators and therapeutic targets in CRC, shaping treatment strategies and patient outcomes. The density, phenotype, and spatial distribution of tumor-infiltrating immune cells (TILs) have been strongly associated with prognosis, particularly in MSI-H CRC. Immunotherapeutic approaches—most notably immune checkpoint inhibition—have yielded significant clinical benefits in MSI-H tumors, while novel strategies such as CAR-T cells and cancer vaccines offer future promise. Additionally, conventional treatments like chemotherapy and radiotherapy influence the immune landscape of the TME, modulating both pro- and anti-tumoral immune responses. This section discusses the prognostic value of immune infiltration and the immunomodulatory effects of conventional therapies in CRC.

### 4.1 Prognostic value of immune cell infiltration

Tumor-infiltrating immune cells, particularly CD8^+^ cytotoxic T lymphocytes, CD4^+^ helper T cells, and NK cells, are associated with favorable prognosis in CRC. High TIL densities correlate with prolonged overall survival (OS), especially in early-stage and MSI-H tumors (Wankhede et al., 2024). The Immunoscore, a quantitative measure of CD3^+^ and CD8^+^ T cell infiltration in tumor regions, has been validated as a robust prognostic biomarker (Zhao et al., 2019; Orhan et al., 2022).

In CMS1, which constitutes 14% of CRC cases, elevated TIL infiltration and the presence of tertiary lymphoid structures (TLS) contribute to an immunologically active TME and improved clinical outcomes (Guinney et al., 2015; Williams et al., 2019; Jung et al., 2022). Conversely, CMS4 and metastatic CRC subtypes are often characterized by low TIL levels and high densities of immunosuppressive cells such as MDSCs and Tregs, correlating with worse prognosis (Koi and Carethers, 2017; Edin et al., 2019; Rahimi et al., 2025).

Natural killer (NK) cell infiltration has also been linked to improved outcomes in MSI-H CRC (Sconocchia et al., 2014); however, their function is often suppressed in late-stage disease due to immunosuppressive factors within the TME. These findings underscore the clinical utility of immune profiling tools like the Immunoscore in refining CRC prognosis and guiding treatment.

### 4.2 Effects of conventional therapies on the immune landscape

Conventional therapies such as chemotherapy and radiotherapy not only act directly on tumor cells but also significantly modulate the immune contexture of the TME. Chemotherapeutic agents like 5-fluorouracil and oxaliplatin induce immunogenic cell death (ICD), facilitating the release of tumor antigens and recruitment of cytotoxic immune cells (Wang et al., 2018; Zhai et al., 2023). Oxaliplatin, in particular, enhances dendritic cell maturation and demonstrates synergy with ICIs in MSI-H CRC (Yan et al., 2024).

However, chemotherapy may also promote the expansion of immunosuppressive populations like MDSCs and Tregs, thereby dampening anti-tumoral immune responses (Bilotta et al., 2022; Wang et al., 2022; Kang and Zappasodi, 2023). Similarly, radiotherapy promotes immune activation through increased antigen presentation and the abscopal effect—where local radiation leads to systemic anti-tumor responses (Bilotta et al., 2022; Kundu et al., 2024; Van Dieren et al., 2024). Clinical studies show promising results for combining stereotactic body radiotherapy with ICIs in metastatic CRC (Kumar et al., 2023; Liu and Chi, 2023; Urias et al., 2025).

Yet, radiotherapy can simultaneously enhance levels of immunosuppressive cytokines such as TGF-β and IL-10, which negatively regulate T cell and NK cell functions (Colton et al., 2020; Bergerud et al., 2024; Liao et al., 2024). Thus, the immune-modulatory duality of these therapies necessitates thoughtful integration with immunotherapy regimens.

Immune infiltration patterns and their modulation by therapies form a critical axis in CRC prognosis and treatment design. While MSI-H CRC benefits significantly from immune-based therapies, MSS subtypes remain a challenge due to TME-induced immune resistance. Tools such as the Immunoscore enhance clinical decision-making by quantifying immune infiltration, and combination therapies that harness conventional treatment-induced immunogenicity with ICIs are increasingly supported by clinical data. Future directions should focus on overcoming immunosuppression in MSS CRC, refining immune biomarkers, and designing synergistic treatment protocols to maximize therapeutic benefit. Due to the limitations of conventional treatments, innovative approaches such as immunotherapy have become more important.

## 5 Immunotherapy strategies in CRC: challenges and opportunities

**FIGURE 4:**
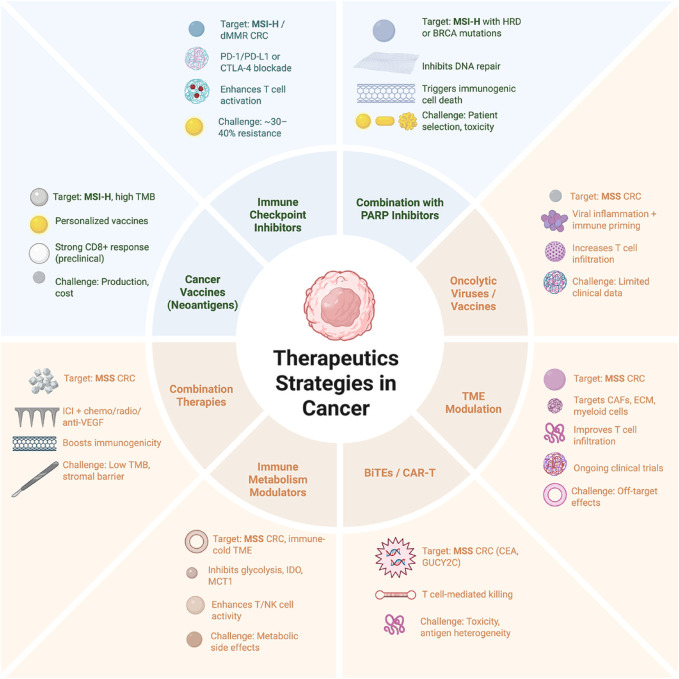
Therapeutic strategies tailored for colorectal cancer according to microsatellite status (Microsatellite instability-High- MSI-H and Microsatellite Stable-MSS). In microsatellite instability-High (MSI-H)/deficient mismatch repair (dMMR) colorectal cancer (CRC), the enhanced immunogenicity enables the success of immune checkpoint inhibitors (ICIs), combination strategies with PARP inhibitors, and neoantigen-based personalized cancer vaccines. These approaches aim to exploit DNA repair deficiencies and tumor antigenicity to boost anti-tumor immune responses. In contrast, MSS CRC, which exhibits low tumor mutational burden and immunologically “cold” microenvironments, requires alternative immune priming approaches. These include combination therapies with chemotherapy, oncolytic viruses and vaccines to induce local inflammation, modulation of the tumor microenvironment (TME) to improve T cell infiltration, metabolic reprogramming to enhance T/NK cell activity, and targeted cellular therapies such as bispecific T cell engagers (BiTEs) and CAR-T cells. Each segment outlines the strategy’s target population, mechanism of action, clinical promise, and current limitations. Together, these approaches reflect a multifaceted effort to overcome immune resistance in CRC and guide precision immunotherapy selection. The image was created with BioRender (https://BioRender.com).

The emergence of immunotherapy has revolutionized the treatment landscape for various malignancies; however, its success in CRC has been largely restricted to specific molecular subtypes (Figure 4). Unlike melanoma or non-small cell lung cancer, where ICIs have become standard of care, most CRC patients do not benefit from current immunotherapeutic approaches due to intrinsic or acquired resistance mechanisms. Understanding the immune contexture of CRC is thus critical for improving patient selection and expanding the effectiveness of immunotherapy (Ganesh et al., 2019).

### 5.1 Immune checkpoint inhibition in MSI-H CRC

The most significant immunotherapeutic breakthrough in CRC has been observed in patients with MSI-H or deficient mismatch repair (dMMR) tumors, which account for approximately 15% of cases. These tumors harbor a high tumor mutational burden, leading to abundant neoantigen production and robust immune cell infiltration (Ganesh et al., 2019). As of 2025, ICIs such as pembrolizumab (anti-PD-1) and nivolumab, with or without ipilimumab (anti-CTLA-4), are integrated into clinical practice in both neoadjuvant and first-line settings for MSI-H/dMMR CRC. In the first-line setting for advanced or metastatic disease, pembrolizumab is approved as monotherapy, demonstrating superior progression-free survival (PFS) compared to chemotherapy (Le et al., 2015; Andr’e et al., 2020). The combination of nivolumab and ipilimumab has also received FDA approval as an initial treatment, yielding durable responses with objective response rates (ORR) of 60%–70% and median overall survival (OS) exceeding 40 months in select cohorts (Overman et al., 2018; Lenz et al., 2022). In the neoadjuvant setting, ICIs have shown remarkable efficacy for localized dMMR/MSI-H CRC. Phase II studies and real-world data indicate exceptional response rates, with single agent pembrolizumab or nivolumab plus ipilimumab achieving pathological complete response (pCR) rates of up to 60%–100% and major pathological responses (MPR), enabling organ preservation and reducing surgical morbidity (Chalabi et al., 2020; Ludford et al., 2023; Cercek et al., 2025). According to updated CSCO guidelines (2025), neoadjuvant immunotherapy induces high pathological response rates in dMMR/MSI-H patients, and integration with chemoradiotherapy improves disease-free survival (DFS) to 80%–90% at 2 years (Chinese Society of Clinical Oncology, 2025). Despite these successes, a subset of MSI-H patients fails to respond to ICIs, suggesting the presence of additional resistance mechanisms. Factors such as Wnt/β-catenin pathway activation, poor antigen presentation, and increased presence of immunosuppressive cells (e.g., Tregs, MDSCs) have been implicated in mediating resistance even in this “hot” tumor context (Ganesh et al., 2019; Grillo et al., 2024). Ongoing efforts are exploring rational combination regimens — such as dual PD-1/CTLA-4 blockade, VEGF inhibition, or radiotherapy-induced immune priming — to further enhance efficacy in MSI-H CRC (Overman et al., 2018; Chalabi et al., 2020).

### 5.2 Immune resistance in MSS CRC

The vast majority of CRC cases (∼85%) are MSS and exhibit low tumor mutational burden, limited T-cell infiltration, and immunosuppressive microenvironments—features collectively referred to as “cold” tumors (Bonaventura et al., 2019). These “cold” tumors exhibit limited T-cell infiltration and generally, fail to respond to ICIs. Multiple mechanisms, including stromal barriers, MDSC accumulation, and defective antigen presentation, contribute to immune exclusion and resistance to ICIs (Bonaventura et al., 2019).

Several strategies are under investigation to overcome these barriers and convert immunologically “cold” MSS tumors into “hot” ones capable of responding to immunotherapy (Figure 4), including the investigational immunotherapy combination of botensilimab (a next-generation anti-CTLA-4 antibody) and balstilimab (an anti-PD-1 antibody) from Agenus Inc., specifically for treating refractory metastatic colorectal cancer (mCRC), particularly microsatellite-stable (MSS) disease. Updated Phase 1b data from the C-800–01 trial (NCT03860272), presented at ESMO GI 2025, reported a median overall survival (OS) of 21 months and a 42% two-year survival rate in MSS mCRC patients without active liver metastases (n = 123) (Bullock et al., 2024; El-Khoueiry et al., 2025). The regimen has been evaluated in over 1,200 patients across Phase 1/2 trials, demonstrating sustained efficacy with objective response rates (ORR) of 20%–25% and progression-free survival (PFS) of 8–10 months in this refractory population (Agenus Inc., 2025; Kwek et al., 2025). With FDA alignment, the combination is advancing toward Phase 3 registration trials by the end of 2025, including combinations with radiation therapy (e.g., NCT07128355) (ClinicalTrials.gov, 2025; Agenus Inc, 2025). Preliminary Phase 2 results highlight its potential to reshape the immunosuppressive TME in MSS subtypes, though challenges such as liver metastasis exclusion and toxicity management persist (Ciardiello et al., 2019; Chalabi et al., 2020).

One promising approach involves combination regimens integrating ICIs with chemotherapy, radiotherapy, anti-angiogenic agents, or MEK inhibitors to enhance antigen presentation and promote immune infiltration (Ciardiello et al., 2019; Chalabi et al., 2020; Wang Y. et al., 2024). Recent preclinical and early-phase clinical data have also highlighted the potential of novel immunotherapy combinations such as botensilimab (an Fc-enhanced CTLA-4 antibody) and balstilimab (an anti-PD-1 agent). This dual checkpoint regimen has demonstrated encouraging activity in refractory MSS mCRC, with durable partial responses and manageable safety in phase I/II trials (El-Khoueiry et al., 2023; Agenus Inc, 2024).

These tumors generally do not respond to ICIs due to insufficient neoantigen load, low expression of immune checkpoints, and the dominance of stromal barriers and regulatory immune populations. Several strategies are under investigation to overcome resistance in MSS CRC (Figure 4):

- *Combination Therapies:* Combining ICIs with chemotherapy, radiotherapy, anti-angiogenic agents (e.g., bevacizumab), or MEK inhibitors to enhance antigen presentation and promote immune infiltration (Ciardiello et al., 2019; Chalabi et al., 2020).
- *Oncolytic Viruses and Vaccines:* Engineered viruses and tumor vaccines aim to convert cold tumors into immunologically active ones by inducing local inflammation and immune priming (Tian et al., 2022; Khosravi et al., 2024; Ma J. et al., 2025; Nadafi et al., 2025a).
- *Modulation of the Tumor Microenvironment:* Targeting CAFs, ECM components, or suppressive myeloid cells to increase T cell access and restore immune responsiveness (Ciardiello et al., 2019).
- *Bispecific Antibodies and CAR-T Cells:* Novel modalities such as bispecific T-cell engagers (BiTEs) or CAR-T cells targeting CRC-associated antigens (e.g., CEA, GUCY2C) are being evaluated in preclinical and early-phase clinical studies (Bonaventura et al., 2019).

Additional therapeutic avenues include oncolytic viruses and tumor vaccines designed to induce local inflammation and immune priming, as well as modulation of the tumor microenvironment via CAF targeting or ECM-degrading strategies to improve T-cell access (Tian et al., 2022; Nadafi et al., 2025b). Bispecific antibodies and CAR-T or CAR-NK cell therapies targeting CRC-associated antigens (CEA, GUCY2C, Claudin-18.2) are emerging tools under clinical evaluation for MSS CRC (Ma N. et al., 2025; Yang et al., 2024).

Collectively, these innovations underscore a shift toward multimodal immunotherapy aimed at re programming the suppressive MSS microenvironment. Future research should focus on identifying predictive biomarkers of response, integrating spatial and single-cell multi-omics data, and rationally combining ICIs with agents that modulate stromal and metabolic barriers to achieve durable benefit in MSS CRC (Lişcu et al., 2024; Wang S. et al., 2024).

### 5.3 Immunoscore and immune-based classification

In response to the observed heterogeneity in CRC immunity, Galon et al. proposed the “Immunoscore”—a quantitative assessment of CD3^+^ and CD8^+^ T-cell densities in the tumor core and invasive margin—which has demonstrated superior prognostic value over traditional TNM staging (Pagès et al., 2018). High Immunoscore values correlate with favorable survival and response to therapy, and efforts are underway to incorporate this system into clinical practice for risk stratification and treatment decision-making (Pagès et al., 2018).

Similarly, transcriptomic studies have identified immune subtypes such as the C4 subtype, enriched in cytotoxic and Th1-type immune cells and characterized by high cytolytic activity, and C2/C3 subtypes, which exhibit angiogenesis, stromal enrichment, and immune exclusion (Soldevilla et al., 2019; Tang et al., 2020; Peng, 2022). These findings highlight the potential for immune-based molecular classifications to guide personalized therapy.

### 5.4 Emerging and future immunotherapeutic approaches in colorectal cancer

To improve the efficacy of immunotherapy in CRC, particularly in MSS tumors, several promising avenues are being explored:

- *Microbiota Modulation:* Preclinical and clinical evidence suggests that modulating the gut microbiota through probiotics, prebiotics, antibiotics, or fecal microbiota transplantation (FMT) can enhance immunotherapy response. Certain bacteria such as *Akkermansia muciniphila* and *Bifidobacterium* have been associated with improved ICI outcomes (Gopalakrishnan et al., 2018).
- *Spatial Transcriptomics and Single-Cell Profiling:* Advanced molecular profiling methods are enabling unprecedented insights into the spatial organization and phenotypic states of immune cells within CRC tumors. These technologies may help identify novel immune targets and resistance mechanisms (Roelands et al., 2017).
- *Personalized Neoantigen Vaccines:* Custom vaccines based on tumor-specific neoantigens are being designed to stimulate strong, patient-specific immune responses (Sahin et al., 2020).
- *Targeting Innate Immune Sensors:* Agonists of TLRs, STING, and other innate immune pathways are being investigated as adjuvants to promote tumor inflammation and T-cell priming (Bayyurt Kocabas et al., 2020; Bhatnagar et al., 2022; Temizoz et al., 2022).

## 6 Conclusion and future perspectives

CRC is a biologically heterogeneous malignancy in which the TME plays a pivotal role in shaping disease progression, immune dynamics, and therapeutic response. Within the CRC immune landscape, diverse innate and adaptive immune cells—including CTLs, natural killer NK cells, DC, Tregs, TAMs, and MDSCs—orchestrate a balance between anti-tumoral immunity and immune escape (Ferkel et al., 2025). This balance is heavily influenced by molecular subtypes of CRC, particularly MSI-H versus MSS tumors, and further modulated by the stromal architecture, ECM, and gut microbiota (Gopalakrishnan et al., 2018).

Robust evidence supports the prognostic and therapeutic significance of tumor-infiltrating lymphocytes (TILs), especially CD8^+^ T cells, with high infiltration correlating with improved outcomes and response to ICIs in MSI-H CRC (Pagès et al., 2018). This has positioned ICIs as a standard of care in this subset. However, the immunologically “cold” nature of most MSS CRCs, characterized by low TIL density and enriched immunosuppressive cell populations, limits ICI efficacy and underscores the need for novel strategies (Bonaventura et al., 2019; He et al., 2024). Combinatorial approaches that integrate ICIs with chemotherapy, radiotherapy, stromal modulation, and microbiome-targeted therapies hold promise for converting cold tumors into immunologically active ones (Zhang et al., 2019a; Petitprez et al., 2020).

Despite these advances, several challenges persist. Methodological heterogeneity across studies, limited insight into underexplored immune populations (e.g., mast cells, B cell subsets), and a predominant focus on advanced-stage disease hinder the development of universally applicable immunotherapies. Furthermore, the lack of standardized biomarkers and small patient cohorts limit clinical translation and reproducibility (Drucker and Krapfenbauer, 2013).

To address these challenges, future research must prioritize high-resolution immune profiling—using tools such as single-cell transcriptomics and spatial analyses—to unravel immune heterogeneity, functional states, and spatial interactions within the CRC TME (Taube et al., 2018; Petitprez et al., 2020; Li J. et al., 2023). Standardization of immunological assessment tools like the Immunoscore, alongside the development of dynamic biomarkers predictive of therapy response, will be essential for guiding patient stratification and therapeutic decision-making (Pagès et al., 2018).

In summary, decoding the complex interplay between immune cells, tumor biology, and the microenvironment is critical for advancing CRC management. An integrated, biomarker-driven, and subtype-informed approach will be key to optimizing immunotherapy outcomes and improving prognosis across all CRC subtypes.
